# Conserved Mechanisms of Tumorigenesis in the *Drosophila* Adult Midgut

**DOI:** 10.1371/journal.pone.0088413

**Published:** 2014-02-06

**Authors:** Òscar Martorell, Anna Merlos-Suárez, Kyra Campbell, Francisco M. Barriga, Christo P. Christov, Irene Miguel-Aliaga, Eduard Batlle, Jordi Casanova, Andreu Casali

**Affiliations:** 1 Cell and Developmental Biology Program. Institute for Research in Biomedicine, Barcelona, Spain; 2 Department of Developmental Biology. Institut de Biologia Molecular de Barcelona, Barcelona, Spain; 3 Oncology Program. Institute for Research in Biomedicine, Barcelona, Spain; 4 Department of Zoology, University of Cambridge, Cambridge, United Kingdom; 5 MRC Clinical Sciences Centre, Imperial College London, London, United Kingdom; 6 Institució Catalana de Recerca i Estudis Avançats, Barcelona, Spain; Ecole Normale Supérieur de Lyon, France

## Abstract

Whereas the series of genetic events leading to colorectal cancer (CRC) have been well established, the precise functions that these alterations play in tumor progression and how they disrupt intestinal homeostasis remain poorly characterized. Activation of the Wnt/Wg signaling pathway by a mutation in the gene APC is the most common trigger for CRC, inducing benign lesions that progress to carcinomas due to the accumulation of other genetic alterations. Among those, Ras mutations drive tumour progression in CRC, as well as in most epithelial cancers. As mammalian and *Drosophila*'s intestines share many similarities, we decided to explore the alterations induced in the *Drosophila* midgut by the combined activation of the Wnt signaling pathway with gain of function of Ras signaling in the intestinal stem cells. Here we show that compound Apc-Ras clones, but not clones bearing the individual mutations, expand as aggressive intestinal tumor-like outgrowths. These lesions reproduce many of the human CRC hallmarks such as increased proliferation, blockade of cell differentiation and cell polarity and disrupted organ architecture. This process is followed by expression of tumoral markers present in human lesions. Finally, a metabolic behavioral assay shows that these flies suffer a progressive deterioration in intestinal homeostasis, providing a simple readout that could be used in screens for tumor modifiers or therapeutic compounds. Taken together, our results illustrate the conservation of the mechanisms of CRC tumorigenesis in *Drosophila*, providing an excellent model system to unravel the events that, upon mutation in Apc and Ras, lead to CRC initiation and progression.

## Introduction

Activating mutations in the Wnt and Ras signaling pathways are common in many epithelial cancers. Colorectal cancer (CRC), for example, usually starts with mutations in APC, a negative regulator of the Wnt signaling pathway [1]–[3] followed, in ∼50% of CRC patients, by the oncogenic activation of K-Ras, an event that correlates with the onset of malignancy [4]. This process can be reproduced in mouse models for CRC, where the conditional deletion of APC in the intestinal epithelium imposes a stem/progenitor-like phenotype, leading to massive crypt hyperproliferation and formation of benign tumors known as adenomas [3], [5]–[7]. In turn, expression of K-Ras gain of function alleles in Apc mutant tumor cells induces the development of large, aggressive adenocarcinomas [7], [8]. However, whereas this genetic events leading to CRC have been well established, the precise genetic programs that these alterations impose, which role play in tumor progression and how they disrupt intestinal homeostasis remain poorly characterized.

It has been shown that the removal of APC specifically in the intestinal stem cells (ISCs), but not in their progeny, leads to the formation of lesions that progress to adenomas in less than three weeks, suggesting that ISCs may represent the cell of origin of CRC [9]. Mammalian ISCs are located at the base of the intestinal crypts, where they self-renew and generate a transient amplifying population. These progenitor cells undergo several rounds of division while moving upwards along the crypt axis and finally arrest and differentiate towards enterocytes, enteroendocrine cells or goblet cells [10]. Interestingly, there are many relevant similarities between mammalian and *Drosophila* intestines [11], [12]. The adult *Drosophila* midgut epithelium is also maintained by a population of ISCs that regenerate the stem cell pool or become quiescent progenitor cells (known as enteroblasts, or EB), which ultimately differentiate towards enterocytes (ECs) or enteroendocrine cells (EEs) [13], [14]. The Wg/Wnt signaling pathway is required for both mammalian and fly intestinal stem cell homeostasis [15]–[18], and its constitutive activation in *Drosophila* through mutations in the APC homologues, Apc and Apc2, results in ISC hyperproliferation and midgut hyperplasia [17]. Moreover, EGFR/Ras signaling pathway activity also promotes ISC division and is therefore required for ISC proliferation [19]–[25].

*Drosophila* has been widely used to recapitulate key aspects of human cancer [26]. Here, we generated clones that combined the loss of Apc with the expression of the oncogenic form of Ras, Ras^V12^. We show that these compound Apc-Ras clones, but not clones bearing the individual mutations, expand as aggressive intestinal tumor-like over-growths that reproduce many hallmarks of human CRC. Of note, similar conclusions about the ability to form tumors upon loss of Apc and oncogenic Ras expression have been reached in an recent study [27]. Moreover, we show that flies bearing Apc-Ras clones suffer a progressive deterioration in intestinal homeostasis, providing a simple readout that could be used in screens for tumor modifiers or therapeutic compounds. Our results show that the mechanisms leading to tumorigenesis in the human colon upon mutation of Apc and Ras are conserved in the *Drosophila* adult midgut, providing an excellent model system to analyze the genetic events involved in tumor initiation and progression.

## Results

### Combination of Apc mutations and oncogenic Ras expression induces the outgrowth of clones in the adult *Drosophila* midgut

The similarities between mammalian and *Drosophila* intestines prompted us to investigate whether the generation of compound Apc-Ras clones in the adult midgut epithelia, mutant for both forms of the *Drosophila* APC gene, *Apc* and *Apc2*, and over-expressing the oncogenic form of Ras, UAS-Ras^V12^ (refs [28], [29]), would reproduce the first steps of CRC. In order to test this hypothesis we induced, by means of the MARCM technique [30], GFP-marked Apc-Ras clones in the ISCs, the only dividing cells in the adult midgut [13], [14]. As controls, we generated *i*) wild type clones, *ii*) clones mutant for *Apc* and *Apc2* (Apc clones) and *iii*) clones over-expressing UAS-Ras^V12^ (Ras clones).

**Figure 1:**
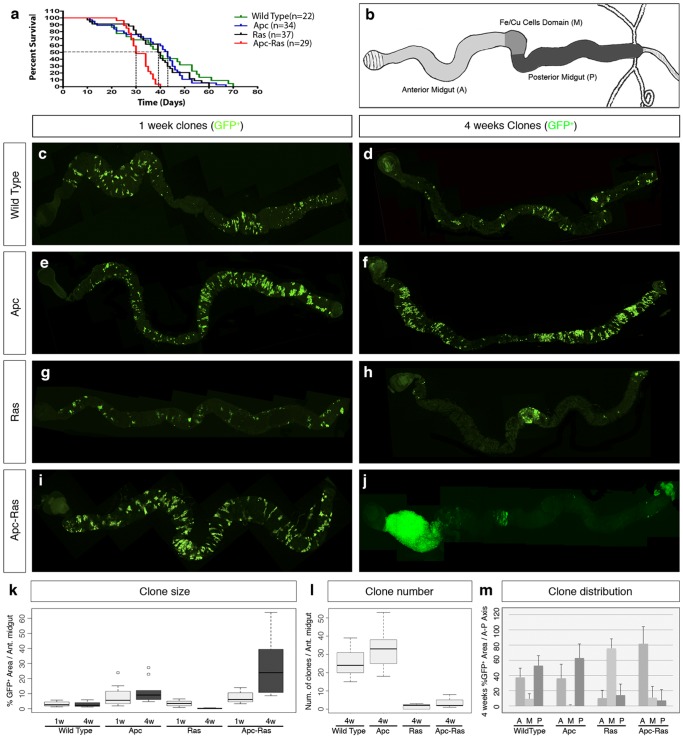
Loss of Apc combined with activated Ras expression has a synergistic activity inducing over-growths in the adult *Drosophila* gut. a, life span of flies bearing wild type, Apc, Ras and Apc-Ras clones. b, diagram of an adult *Drosophila* midgut. A, M, and P mark the areas used to analyze clone distribution along the anteroposterior axis (m). c–j, adult midguts showing wild type, Apc, Ras and Apc-Ras clones marked by GFP (green) one and four weeks after induction. k, box-plot graph of clone area (GFP^+^) per anterior gut area one and four weeks after clone induction. l, box-plot graph of the total number of clones in the anterior gut four weeks after clone induction. m, histogram of the clone area (GFP^+^) distribution along the anteroposterior axis four weeks after induction.

**Figure 2:**
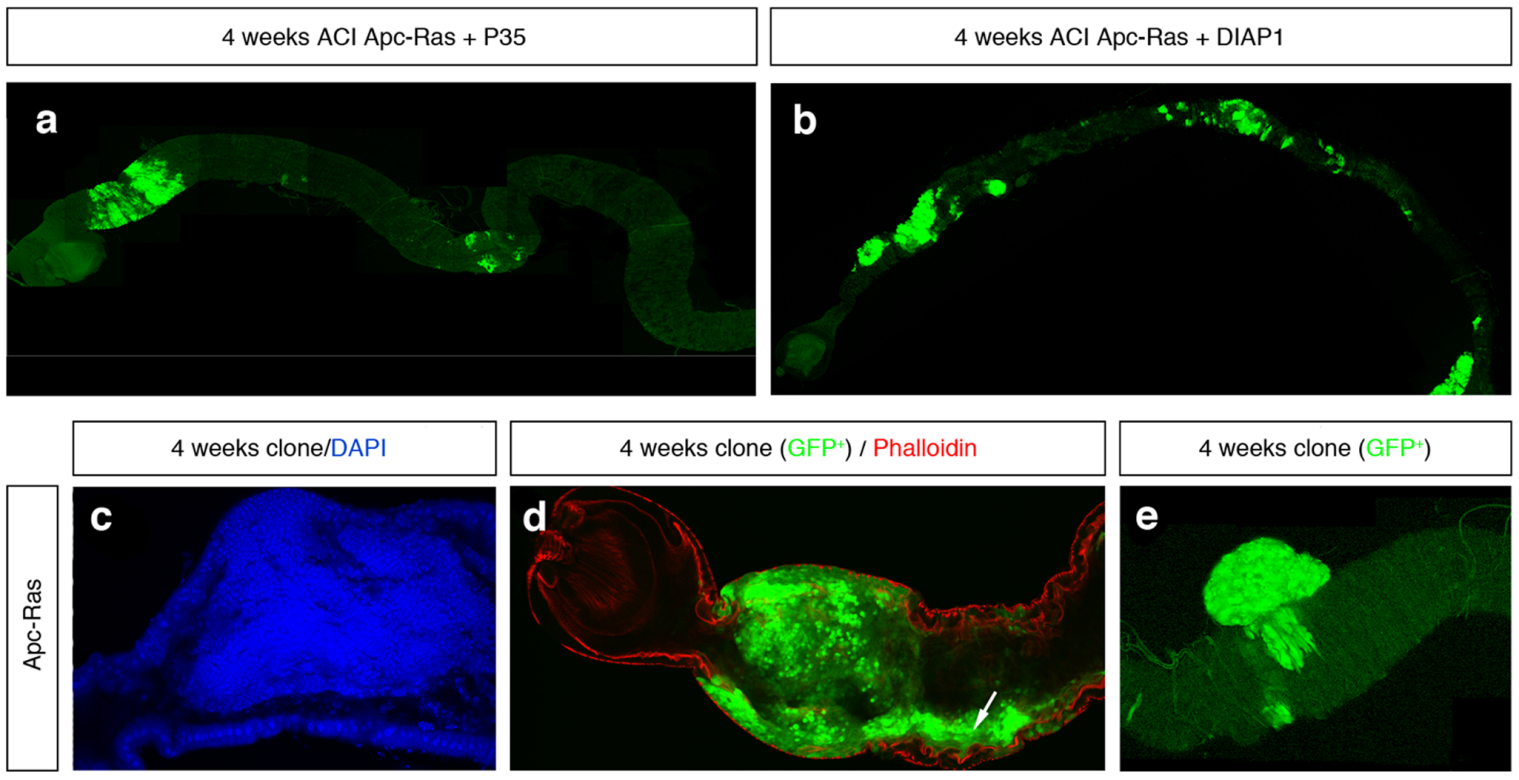
The Ras^V12^-driven tumor suppressor mechanism does not depend on apoptosis. **a–b**, adult midguts bearing Apc-Ras clones over-expressing the anti-apoptotic transgenes **a**, UAS-P35 (Apc-Ras-UAS P35) or **b**, UAS Diap1 (Apc-Ras-UAS Diap1) four weeks after clone induction. **c–e**, Apc-Ras clones four weeks after induction detected by DAPI (**c**, blue) or GFP (**d–e**, green). Apc-Ras clones invade most of the luminal space (**c**), invade the surrounding wild type epithelium (arrow), marked on its apical side by phalloidin (red) (**d**) and, in some cases, grow towards the muscular layer (**e**).

We first noticed that flies bearing Apc-Ras clones had a shorter life span compared to flies bearing control clones, displaying a ∼50% survival rate four weeks after clone induction (Figure 1A). We also observed that, one week after clone induction, Apc-Ras and control clones showed relatively small differences in clone size, number and distribution along the anterior and posterior midgut (Figure 1B,C,E,G,I,K and Table 1). Four weeks after clone induction, wild-type clones did not change noticeably (Figure 1D,K and Table 1) and Apc clones slightly increased in size but remained similar in number and distribution (Figure 1F,K,L,M and Table 1). However, most Apc-Ras clones disappeared, but the few remaining were dramatically enlarged and usually localized in the anterior-most part of the anterior midgut (Figure 1J,K,L,M and Table 1). In contrast, Ras clones also disappeared but the few remaining were small and localized mostly in the central region of the gut (M) (Figure 1B,H,L,M and Table 1), suggesting the existence of a mechanism able to induce the disappearance of Ras^V12^-expressing cells. This mechanism of clone decline does not rely on apoptosis, as the over-expression of the anti-apoptotic viral protein P35 or the apoptosis inhibitor Diap1 did not noticeably increase the number of Apc-Ras clones (Figure 2A,B).

**Table 1:** Clone characterization.

Genotype	W	n	Number of clones (*num clones ± SD*)			Clone size (*GFP^+^ area/domain area ± SD*)			Clone distribution (*GFP^+^ area/total area ± SD*)		
			A	M	P	A	M	P	A	M	P
Wild Type	1	8	37±10	2±2	58±13	3±2	0,1±0,1	3±2	49±10	0,3±0,3	51±10
Apc	1	10	30±7	6±4	40±14	8±7	2±1	11±5	32±12	2±1	66±12
Ras	1	9	15±5	5±2	16±10	3±2	16±15	3±3	39±19	31±19	31±23
Apc-Ras	1	10	23±8	5±3	23±6	7±4	8±9	4±2	52±15	10±9	38±18
Wild Type	4	10	25±7	8±4	24±4	3±1	4±3	4±2	38±12	9±7	53±13
Apc	4	10	33±10	7±5	37±6	11±8	2±2	15±7	36±19	0,7±0,8	63±18
Ras	4	12	2±1	2±1	2±2	0,2±0,2	12±7	0,4±0,5	10±10	76±12	14±14
Apc-Ras	4	19	3±2	2±2	3±3	26±16	19±25	3±5	82±22	11±15	7±14

Quantification of the number, size and distribution of the clones of the different genotypes analyzed. (A) anterior midgut, (M) Fe/Cu domain, (P) posterior midgut, (W) weeks after clone induction, (n) number of guts analyzed.

**Figure 3:**
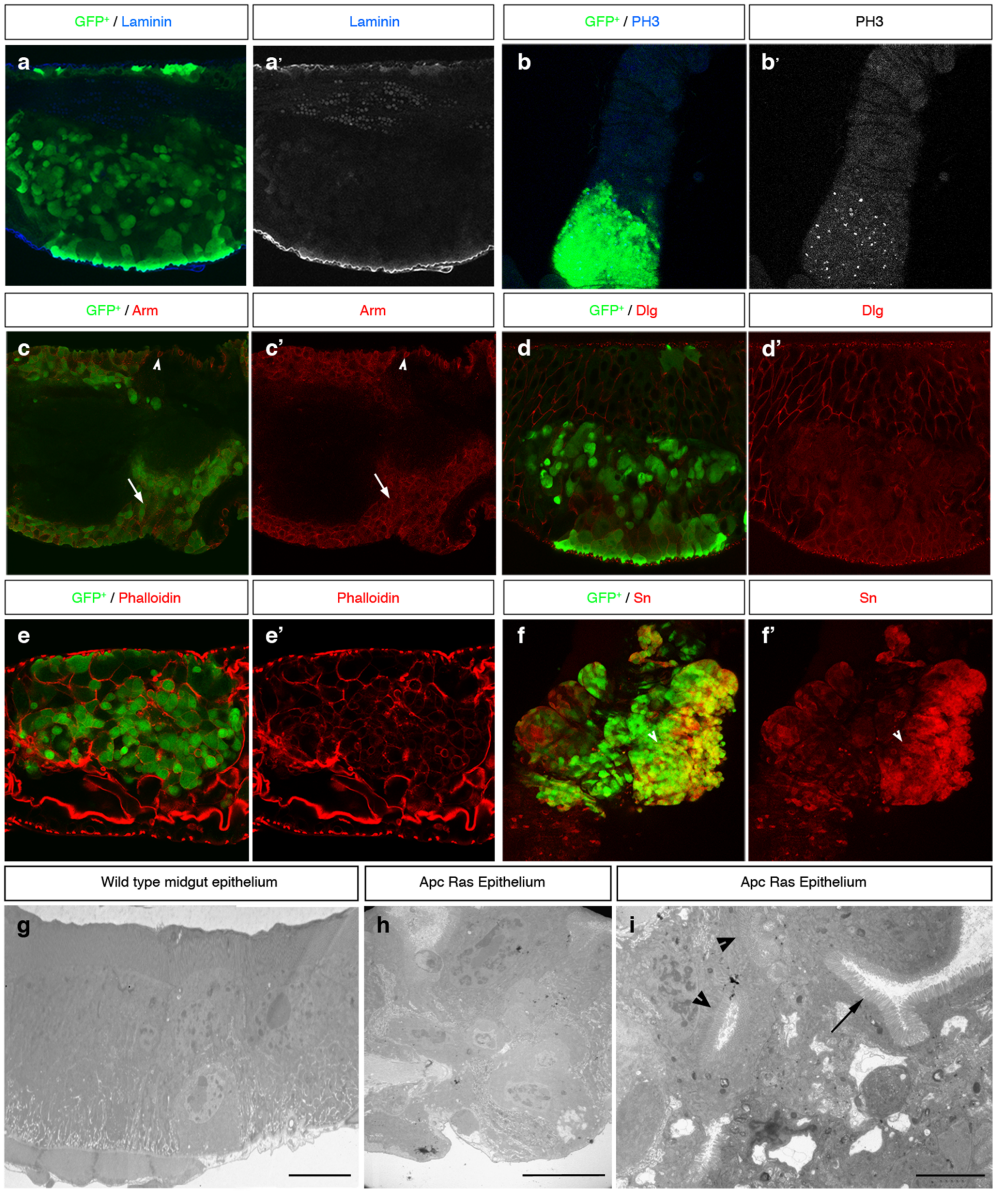
Apc-Ras clones (green) show tumor-like characteristics. **a**, Apc-Ras clones do not break the basal lamina, marked by laminin B (blue). **b**, Apc-Ras clones show a high number of mitotic cells marked by PH 3 staining (blue). **c**, Arm (red) is expressed at high levels in Apc-Ras clones (arrow). In neighbouring wild type ISCs Arm does not localize in the domain that contacts the basal lamina (arrowhead). **d**, The basolateral marker Dlg (red) is delocalized from the membrane in Apc-Ras clones. **e**, The actin binding protein Phalloidin, normally accumulated in the apical side of the enterocytes, shows heterogeneous and delocalized expression in Apc-Ras clones. **f**, Expression of the tumoral marker Singed (red) in Apc-Ras clones. **g**, Electron micrograph of an wild type midgut epithelium. **h**, Electron micrograph of an Apc-Ras clone showing non-polarized cells located far from the basal lamina (arrowhead) and aberrant tissue organization, bar 10 µm. **i**, Electron micrograph of an Apc-Ras clone showing multiple lumens (arrowheads), bar 5 µm. L, lumen. M, muscle.

We also observed that Apc-Ras clones, but not control clones, occupied almost all the luminal space (Figure 2C), colonized the surrounding wild type intestinal epithelium (Figure 2C, arrow) and, in 17% of the cases (n = 117), grew below the wild type epithelia, suggesting invasive abilities (Figure 2E), although we were not able to detect any disruption of the basal membrane (Figure 3A). We conclude that loss of Apc combined with activated Ras expression has a synergistic effect able to induce “tumor-like” over-growths to an extent never achieved by clones generated individually, reproducing the initial events in the adenoma to carcinoma transition of CRC.

### Apc-Ras clones show many tumoral hallmarks

Further characterization of Apc-Ras clones showed that they display many tumoral characteristics. We first found that they had an enhanced proliferation rate, with a remarkable increase in the number of cells positive for the mitotic marker phospho-histone 3 (PH 3) compared to the surrounding wild type epithelium (Figure 3B). As expected, Armadillo (Arm), the *Drosophila* homolog of β-catenin, was homogeneously enriched in all Apc-Ras cells (Figure 3C), reflecting the constitutive activation of the Wg signaling pathway. Moreover, Arm staining around the membrane in Apc-Ras cells (Figure 3C, arrow), which differs from the apicolateral staining in adjacent normal ISCs (Figure 3C, arrowhead), indicated loss of cell polarity. This loss of polarity was confirmed by the delocalization of the septate junction marker and neoplastic tumor suppressor Discs large (Dlg) [31] (Figure 3D). We also observed an aberrant tissue organization, as small fragments of the actin-rich microvilli present on the apical, luminal domain of the ECs were not correctly oriented with respect to the apico-basal axis of the midgut (Figure 3E, arrow). Electron microscope analysis of a wild type midgut showed that is lined by a simple epithelium where each cell, including the ISCs, stands on the basal lamina (Figure 3G), as previously described [32]. In contrast, Apc-Ras clones showed an aberrant, dysplastic, highly disorganized tissue with multiple lumens and several layers of non-polarized cells that did not stand on the basal lamina (Figure 2H,I). Finally, Apc-Ras clones, but not control clones, expressed the tumoral marker Singed (Sn) (Figure 2F and Figure S1A). Sn is the *Drosophila* homolog of the actin-bundling protein Fascin1 (ref [33]), a key component of filopodia that has been identified as a target of the β-catenin-TCF signaling in CRC cells and is over-expressed in the invasive front during tumor progression [34]. Interestingly, Sn expression was also localized at the edge of the clones (Figure S1B). In summary, our results show that, four weeks after induction, Apc-Ras clones display tumor-like characteristics. Thus, the synergistic action of loss of Apc and activated Ras is enough to transform normal intestinal epithelia to “tumor-like” over-growths.

### Apc-Ras clones display cell heterogeneity

**Figure 4:**
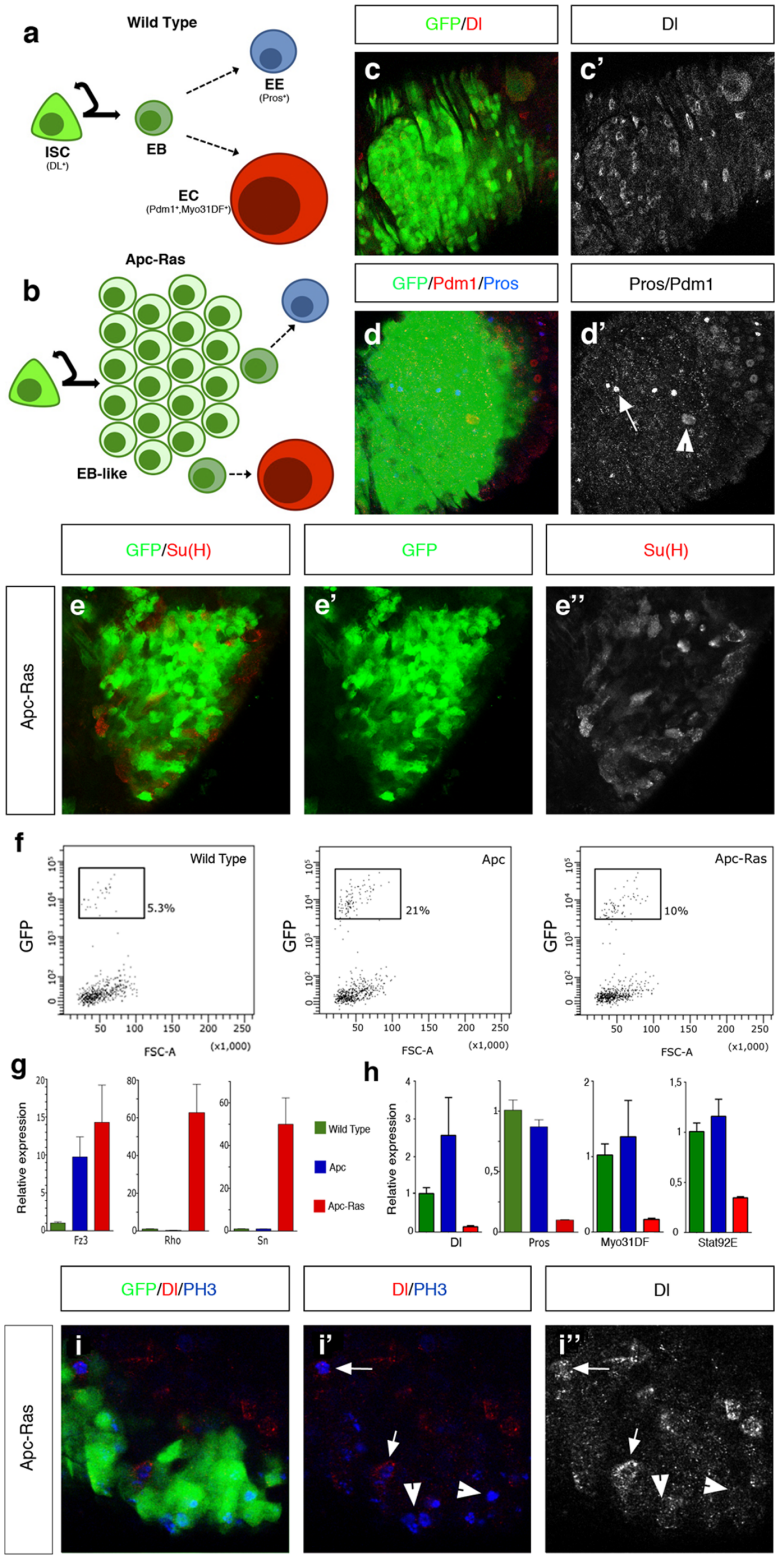
Apc-Ras clones (green) show cell heterogeneity. **a**, Diagram of the normal adult midgut division-differentiation process. **b**, Diagram of Apc-Ras clones, mainly composed by an “EB-like” population that fails to differentiate. **c–e**, Apc-Ras clones contain few ISCs, marked by Dl (red) (**c**), EE cells, marked by Pros (blue, arrow) and ECs, marked by Pdm1 (red, arrowhead) (**d**) and EBs, marked by Su(H)mCherry (red) (**e**). **f**, Graphs of the clone (GFP^+^) population four weeks after clone induction sorted by FACS and used for qRT-PCR analysis. **g**, Validation of the sorting strategy. qRT-PCR analysis shows the increase of the Wg target gene Frizzled3 (Fz3) expression in Apc and Apc-Ras clones, and the increase of the EGFR/Ras pathway target gene Rhomboid (Rho) and the tumoral marker Sn only in Apc-Ras clones. **h**, qRT-PCR analysis showing the decrease in Apc-Ras clones of the ISC marker Dl, the EE cell marker Pros, the EC marker Myo31DF and the JAK/STAT transcription factor stat92e. Note that, as expected, Dl expression is up-regulated in Apc clones due to the increase in the ISC number upon Wg pathway activation. **i**, Staining of Apc-Ras clones with the ISC marker Dl (red) and the mitotic marker PH 3 (blue). Note that most dividing cells do not show Dl staining (arrowhead). In contrast, dividing cells outside the clone show high levels of Dl staining (arrow).

We next analyzed the cellular composition of Apc-Ras clones. Similar to human CRC, which recapitulates the cell hierarchy of the normal intestinal mucosa [35], [36], we detected in Apc-Ras clones cells with phenotypes reminiscent to ISCs, marked by Delta (Dl) (Figure 4A,C), EEs, marked by Prospero (Pros) (Figure 4A,D, arrow), ECs, marked by the POU domain protein 1 (Pdm1) (Figure 4A,D, arrowhead) and EBs, marked by the expression of Su(H)mCherry (Figure 4E). We noticed, however, that most cells within Apc-Ras clones were negative for ISC, EB and differentiation markers (Figure 4C,D,E). In order to further characterize this phenotype, we sorted cells by flow cytometry from wild type, Apc and Apc-Ras clones four weeks after clone induction (Figure 4F). Analysis of expression levels by qRT-PCR showed that the Wg/Wnt target gene Frizzled3 (Fz3), the Ras target gene Rhomboid (Rho) and Sn were expressed as expected (Figure 4G), validating our sorting strategy. qRT-PCR analysis also showed a decrease in the RNA levels of Dl, Pros and the EC marker Myosin31DF (Myo31DF) in Apc-Ras clones (Figure 4H), confirming that many cells within these clones must belong to a new cell type that do not express any of the known cell markers. As the qRT-PCR analysis also showed that the JAK/STAT pathway transcription factor stat92e, required for EB differentiation [37]–[39], was down-regulated in Apc-Ras clones (Figure 4H), we considered the possibility that this new cell type could be formed by EBs that fail to differentiate and named them “EB-like” cells (Figure 4B).

In normal tissue, ISCs fuel growth through accelerated division rates and/or predominance of symmetric division [40]. As there were few ISCs in Apc-Ras clones (Figure 4C,H), we considered the non-mutually exclusive possibility that the proliferation of this new “EB-like” cell population could also account for the growth of Apc-Ras clones. In order to address this issue, we stained Apc-Ras clones with the mitotic marker phospho-histone 3 (PH 3). We observed that most PH 3^+^ cells within the clones showed a very low or non detectable Dl staining (Figure 4I, arrowheads), characteristic of EBs [41], in contrast to the surrounding wild type epithelia where PH 3 staining colocalized with the high Dl staining characteristic of ISCs (Figure 4H, arrow). Therefore, our results suggests that the “EB-like” population in Apc-Ras clones could be formed by EBs that failed to differentiate and acquired the capacity to proliferate, possibly accounting for the massive growth of Apc-Ras clones.

### Gut physiology is altered in flies bearing Apc-Ras clones

**Figure 5:**
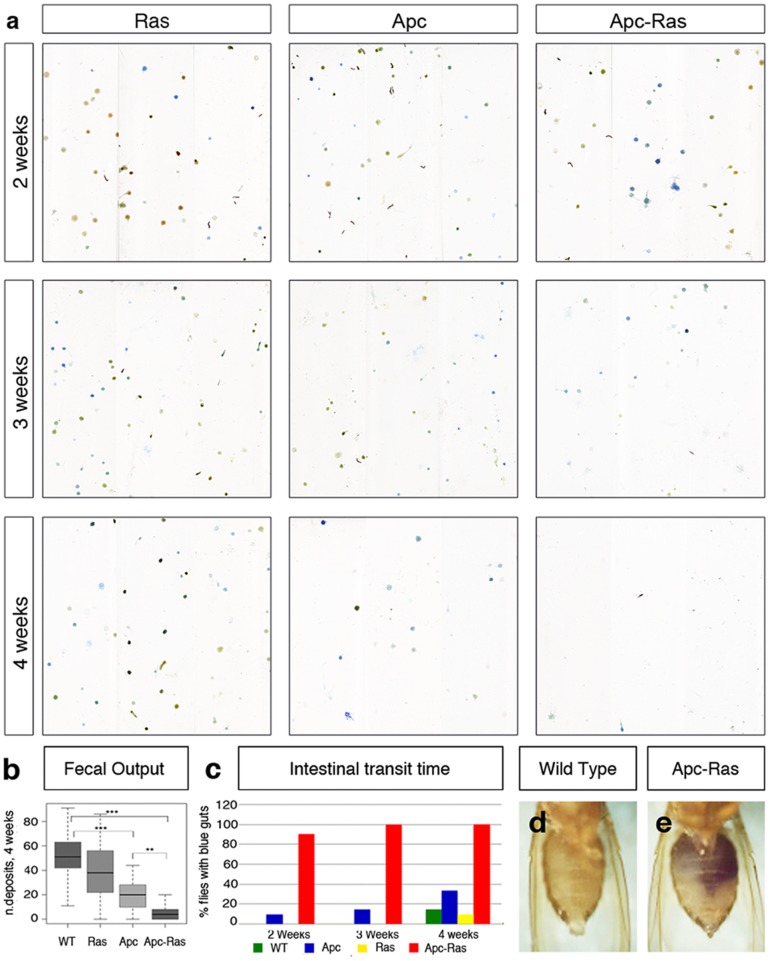
Apc-Ras clones affect gut physiology. **a**, Flies bearing Apc-Ras clones show a decline in the number of fecal depositions. **b**, box-plot showing the reduction in the number of fecal deposits four weeks after clone induction. **c**, Graph showing the number of flies that retain bromophenol blue in their intestines after 72 h of being exposed to a bromophenol blue-free diet. **d–e**, Abdomen of a wild type fly (**d**) or a fly bearing Apc-Ras clones four weeks after induction (**e**) after 72 h of being exposed to a bromophenol blue-free diet.

Since Apc-Ras clones occupy most of the intestinal lumen, we wondered whether gut physiology would be affected. Using a recently developed quantitative assay [42], we observed a severe decrease in the fecal output of flies bearing Apc-Ras clones (Figure 5A,B). A time-course analysis revealed that this reduction was progressive and associated with tumor development, given that four weeks after induction flies were much more severely affected than younger flies (Figure 5A). The reduction in fecal output was preceded by an increase in intestinal transit time, as two days after feeding control flies with blue food their guts appeared “clean” (Figure 5C,D), in contrast to flies bearing Apc-Ras clones, whose guts remained blue for up to a week (Figure 5C,E). We also observed that while flies with Apc clones only displayed an obvious phenotype four weeks after induction, flies with Apc-Ras clones showed reduced intestinal transit just two weeks after induction, and a severe reduction in fecal output three weeks after induction (Figure 5C). These results show that the tumor burden affects the intestinal physiology of the flies and provides a simple strategy for drug screening in CRC treatment as the correlation between this phenotype and tumor severity provides a simple readout for tumor modifiers and/or potential therapeutic compounds.

## Discussion

The development of human CRC is a multistep process, characterized by the accumulation of mutations in different oncogenes and tumor suppressor genes, which lead to the progression from normal mucosa to carcinoma. In this paper we demonstrate that activation of the Wg/Wnt and Ras signaling pathways, the two main players in the onset of human CRC, generate tumors in the *Drosophila* adult midgut. Remarkably, these tumors reproduce many of the hallmarks of human CRC, such as increased proliferation, loss of cell differentiation and polarity, cell heterogeneity, aberrant cell organization and expression of tumoral markers. Taken together, our results show that *Drosophila*, an organism widely recognized by the amenability of its genetics, which has already been used to model human diseases [12], [26], [43], is an excellent system to understand basic genetic questions about human CRC progression.

Surprisingly, we have also found out that the *Drosophila* CRC model shows characteristics that go beyond genetic interactions. For example, the majority of human gastrointestinal tumors are localized in the colon and rectum, yet most of the genetically engineered mouse models of CRC manifest almost exclusively lesions in the small intestine [44]. These results suggest the existence of intrinsic genetic differences along the gastrointestinal tract that render cells refractory to tumor formation and/or to tumor suppression mechanisms in specific regions. Remarkably, we have identified regional differences in *Drosophila*, where Apc-Ras clones tend to develop in the most anterior part of the anterior region. As it has been recently described the existence of a regional organization with distinct morphological and genetic properties along the anteroposterior axis of the adult *Drosophila* midgut [45], [46], its likely that factors regulating the survival of Apc-Ras clones in the anterior midgut will be identified in the near future.

Moreover, Ras clones are eliminated from the anterior and posterior midgut four weeks after clone induction through an apoptosis-independent process, suggesting the existence of a Ras^V12^-driven tumor suppression mechanism. Expression of Ras^V12^ in a single epithelial cell within a monolayer of wild type cells has been shown to induce its apical extrusion or basal protrusion [47], while Ras^V12^ expression in ECs is sufficient to promote their delamination from the epithelia [21], suggesting that similar events may take place in ISCs upon Ras^V12^ expression. Alternatively, Ras^V12^ may induce tumor suppression by promoting cellular senescence, as described in yeast and mammals [48]. Due to the similarities in the signaling pathways controlling intestinal development both in mammals and *Drosophila*, we envisage that elucidation of the mechanism allowing Ras^V12^ clones to bypass tumor suppression will be of importance to understand the regional differences in tumor development along the gastrointestinal tract.

## Materials and Methods

### Clone generation

MARCM clones were generated by a 1 hr heat shock at 37°C of 2–5 days old females and were marked by the progenitor cell marker escargot (esg) Gal4 line driving the expression of UAS GFP. Although esg is expressed only in the progenitor cells (ISCs and EBs), the amount of GFP produced in our experimental conditions was enough to be detected also in EEs and ECs.

### Genotypes

yw UAS flp; esg Gal4 UAS-GFP/CyO; FRT82B Gal80/TM6b flies were crossed with yw hsp70-flp;UAS-Ras^V12^/CyO; FRT82B/TM6b flies to generate control and Ras clones, and were crossed to yw hsp70-flp;UAS-Ras^V12^/CyO; FRT82B Apc2^N175K^Apc^Q8^/TM6b flies to generate Apc and Apc-Ras clones. yw hsp70-flp; esg Gal4 UAS-GFP UAS-Ras^V12^/CyO; FRT82B Gal80/TM6b were crossed with yw hsp70-flp; Sp/CyO; FRT82B Apc2^N175K^Apc^Q8^/TM6b or yw hsp70-flp; Sp/CyO; Su(H)mCherry FRT82B Apc2^N175K^Apc^Q8^/TM6b to generate the Apc-Ras clones described in Figure 4. Apc2^N175K^ is a loss-of-function allele, Apc^Q8^ is a null allele, and UAS-Ras^V12^ is a gain-of-function transgene. Stocks were obtained form Bloomington Stock Center and VDRC. The Su(H)mCherry stock was a gift from Sarah Bray.

### Staining and antibodies

Adult female flies were dissected in PBS. All the digestive tract was removed and fixed in PBS and 4% electron microscopy grade paraformaldehyde (Polysciences, USA) for 35 minutes. Samples were rinsed 3 times with PBS, 4% BSA, 0.1% Triton X-100 (PBT-BSA), incubated with the primary antibody overnight at 4°C and with the secondary antibody for 2 hours at room temperature. Finally, the samples were rinsed 3 times with PBT-BSA and mounted in DAPI-containing media (Vectashield, USA). All the steps were performed without mechanical agitation. Primary antibodies mouse α-Pros (1:100), α-Dl (1:10), α-Arm (1:10), α-Dlg (1:250) and α-Sn (1:50) were obtained from the Developmental Studies Hybridoma Bank (DSHB), rabbit α-PH 3 (1:100) from Cell Signalling (USA), rabbit α-Pdm1 (1:1000) was a gift of Dr.Yang Xiaohang (Institute of Molecular and Cell Biology, Singapore), rabbit α-laminin (1:1000) was from Sigma (USA) and goat α-GFP (1:500) was form Abcam (UK). Secondary antibodies were from Invitrogen (USA). TRICT-conjugated Phalloidin (Sigma, USA) was used at 5 µg/ml. Images were obtained on a Leica SPE or Leica SP5 confocal microscopy and processed in Photoshop CS5 (Adobe, USA).

### Quantifications

GFP area was calculated using ImageJ 1.34 s (Rasband, W.S., http://rsb.info.nih.gov/ij/). Statistical analysis was performed applying the Wilcoxon Signed-Ranks Test. Differences were considered significative when p<0.05.

### Clone gene expression analysis

*Drosophila* midguts were dissected from control, Apc or Apc-Ras flies four weeks after induction. In order to obtain single cell suspensions, samples were incubated in PBS containing 0,4 mg/ml dispase (Gibco) for 15 min at 37°C, syringed using a 27 G needle and washed twice with PBS. After selecting for the viable population (propidium iodide negative), GFP^+^ cells were isolated by fluorescence activated cell sorting (FACS) using a FACSAria 2.0 (BD Biosciences) and RNA was extracted and amplified as recently described [49]. Gene expression levels were assessed using Power Sybr Green quantitative PCR (Applied Biosystems) following the manufacturer's instructions. Actin 5C was used as an endogenous control for normalization and differences in target gene expression were determined using the StepOne 2.0 software (Applied Biosystems). All measurements were performed in triplicate from three independent sorting experiments. Primer pairs for each gene were the following:

Dl forward) GAGCAAGTCGAGTGTCCAAA

Dl reverse) GCACCAACAAGCTCCTCAT

Fz3 forward) TGCTCTGCTCGTCTCTGTTT

Fz3 reverse) ATGCACAACTCGTGCTTCTC

Myo31DF forward) GATCCAGGTGAAGAGACGGT

Myo31DF reverse) TTCCAAACCATTGAGGATGA

Pro forward) CCGATGATCTGTTGATTGCT

Pro reverse) GGTACCTCAATGTGGTTATTGC

Rho forward) CTCCATCATTGAGATTGCCA

Rho reverse) GACGATATTGAAGCCCAGGT

Sn forward) AGGATCTGCATAAGATCGCA

Sn reverse) CAGCTGCGTGGATCAATAAT

Stat92e forward) GTTGCGACTGGATCTCTGAC

Stat92e reverse) ATCTGCTTGAACAGGTGCAG

### Intestinal physiology assays

Defecation assays were performed and quantified as previously described [42]. At least 20 flies of each genotype were monitored over the course of four weeks in fly vials with standard food at 25°C. Once a week, they were individually transferred to clear plastic cuvettes containing the same food supplemented with 0.5% bromophenol blue for 72 hours. Digital images of the cuvette walls were obtained using an Epson Perfection 4990 Photo scanner. After each defecation assay, flies were transferred to food without the dye in order to monitor the dye's intestinal transit by visually monitoring the dye's clearance time.

## Supporting Information

Figure S1 **Expression of the tumoral marker Singed (Sn).** a, Wild type, Apc and Ras clones (green) four weeks after clone induction do not express Sn (red). Sn is only expressed in the tracheal cells that surrounds the intestinal epithelia. **b**, In Apc-Ras clones (green) four weeks after clone induction, Sn (red) is expressed on the clone edge.
(TIF)
