# Supplementary figures and images for: Conserved Mechanisms of Tumorigenesis in the Drosophila Adult Midgut
Source: PLoS One. 2014 Feb 6;9(2):e88413. doi: 10.1371/journal.pone.0088413 (PMC3916428; doi:10.1371/journal.pone.0088413)

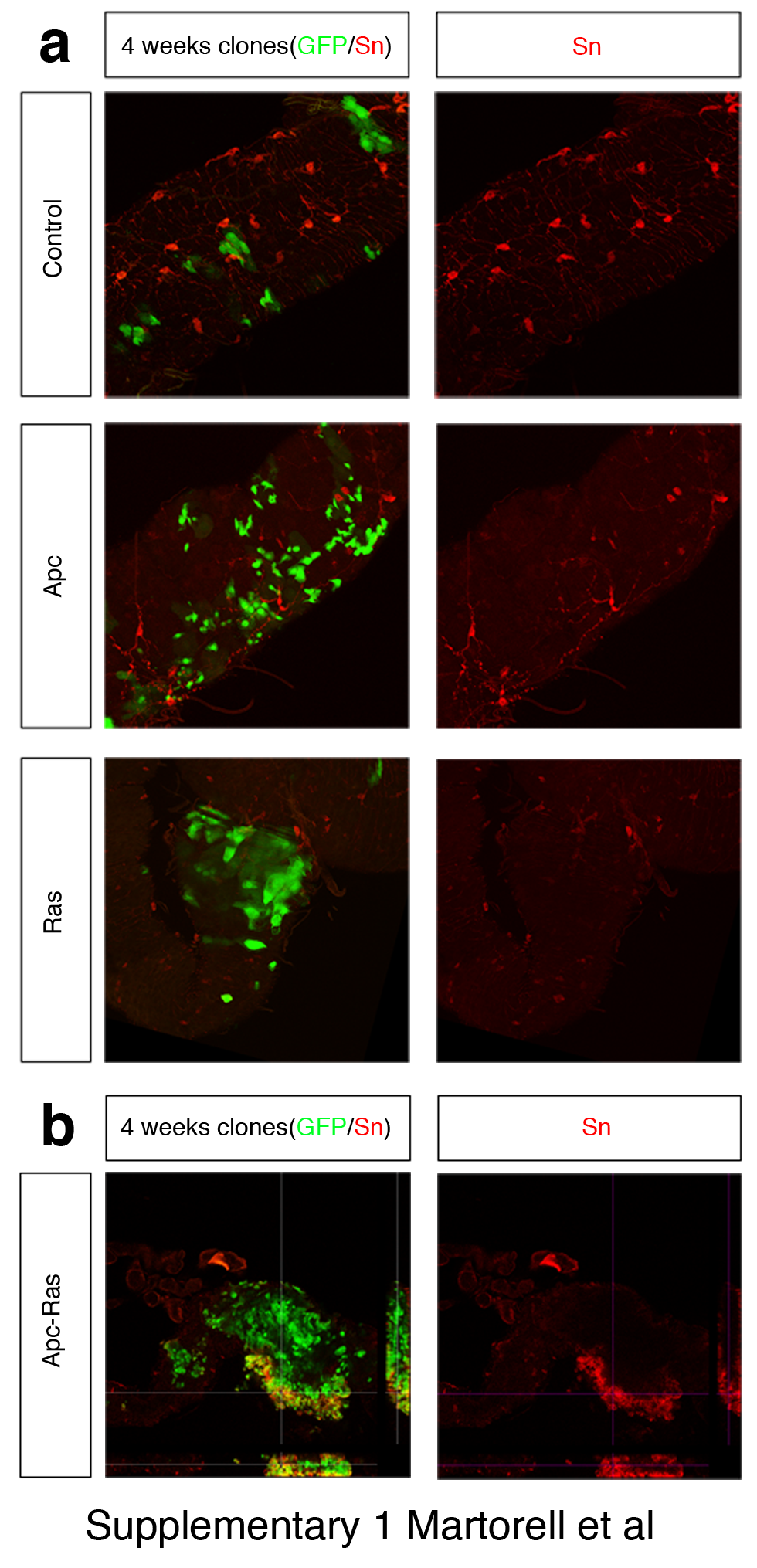

Supplement: Figure S1 — Expression of the tumoral marker Singed (Sn). a, Wild type, Apc and Ras clones (green) four weeks after clone induction do not express Sn (red). Sn is only expressed in the tracheal cells that surrounds the intestinal epithelia. b, In Apc-Ras clones (green) four weeks after clone induction, Sn (red) is expressed on the clone edge. (TIF) [file pone.0088413.s001.tif]
